# Trends in obesity, leisure-time physical activity, and sedentary behavior in Korean adults: Korea national health and nutritional examinations survey from 2014 to 2021

**DOI:** 10.1371/journal.pone.0296042

**Published:** 2024-01-03

**Authors:** Yunmin Han, Hoyong Sung, Younghwan Choi, Yeon Soo Kim

**Affiliations:** 1 Seoul National University, Seoul, Republic of Korea; 2 Department of Military Kinesiology, Korea Military Academy, Seoul, Republic of Korea; 3 Institute of Sports Science, Seoul National University, Seoul, Republic of Korea; Severance Hospital, Yonsei University College of Medicine, REPUBLIC OF KOREA

## Abstract

**Objectives:**

This study aimed to investigate trends in obesity by dividing it based on body mass index (BMI) and waist circumference indicators, sedentary behavior, and leisure-time physical activity (LTPA) in Korean adults from 2014 to 2021. This study also aimed to determine the adherence rate of people with obesity to physical activity.

**Methods:**

Data from the Korea National Health and Nutritional Examination Survey (KNHANES) from 2014 to 2021 were used. A total of 42,676 participants 19 years or older were included in the final analysis. Sociodemographic characteristics, anthropometric measurements, and physical activity levels were recorded. Physical activity levels were assessed using the Global Physical Activity Questionnaire, a self-reported questionnaire. Multivariable logistic regression analysis adjusted for covariates was used to investigate the prevalence of obesity and sitting time or adherence to meeting the physical activity guidelines for each survey year.

**Results:**

This study included 42,676 adults. The weighted prevalence of obesity in all ages significantly increased from 30.8% (29.1%-32.5%) in 2014 to 34.5% (32.9%-36.2%) in 2017 and 37.3% (35.5%-39.1%) in 2021 (*p* for trend < 0.004). The weighted adherence rate to LTPA ranged from 25.5% (95% confidence interval [CI], 23.7%-27.2%) in 2014 to 20.5% (95% CI, 18.7%-22.2%) in 2021(*p* for trend < 0.001). The weighted prevalence of sitting time for 8 h/day or more significantly increased from 46.7% (44.4%-49.0%) in 2014 to 56.2% (54.4%-58.0%) in 2017 and 63% (60.7%-65.3%) in 2021 (*p* for trend < 0.001). According to this study, the LTPA level among women with obesity was significantly low.

**Conclusion:**

From 2014 to 2021, obesity and sedentary behavior significantly increased and adherence to LTPA decreased among Korean adults. Given these concerning trends, comprehensive interventions are needed at the national level to encourage healthy lifestyle behaviors.

## Introduction

The prevalence of non-communicable diseases (NCDs) has rapidly increased worldwide [1, 2]. In addition, there has been an increase in the prevalence of NCDs such as obesity and sedentary lifestyle in the Korean population, imposing a greater social burden [3, 4]. The global prevalence of obesity is on an increasing trend, and the prevalence of central obesity, a major risk factor for chronic diseases, was reported at 41.5% [5]. In contrast, physical activity levels, a key factor in preventing obesity, have generally declined [6], and sedentary behavior has increased significantly within the past 10 years [7]. Increased sedentary behavior and decreased physical activity have been identified as risk factors for obesity, emphasizing the need to enhance dietary habits and environmental conditions and increase physical activity to combat this issue [8].

The World Health Organization (WHO) recommends that adults perform at least 150–300 minutes of moderate-intensity or 75–150 minutes of vigorous-intensity aerobic physical activity per week [9]. The U.S. recently showed an adherence rate of > 60% every year for 150 minutes of moderate-to-vigorous physical activity, and the adherence rate of physical activity in East and Southeast Asia was approximately 82.7% [10, 11]. However, over the past five years, the adherence rate for physical activity in Korea has remained < 50%, which has significantly decreased yearly [12].

In recent years, several countries have conducted domain-specific assessments of physical activity levels [13, 14]. While it is widely known that the level of physical activity has a positive impact on health-related variables, its effects on health may differ for leisure time physical activity (LTPA) and occupational physical activity [15–17]. For effective strategies aimed at promoting public health, it is imperative to differentiate between LTPA and occupational physical activity [18]. In addition, LTPA, in which individuals voluntarily participate, plays an essential role in maintaining physical health, as well as in socialization, communication, maintaining interpersonal relationships, and dealing with challenges [19, 20].

The latest guidelines for physical activity highlight both the adherence rate to physical activity and the suggested duration of sedentary behavior [9, 21]. Over the last two decades, research has indicated that high levels of sedentary behavior are associated with adverse health outcomes [22–24], and it has recently attracted increasing attention as a public health issue [25, 26]. In a recent meta-analysis, a high level of moderate-intensity physical activity was found to be necessary to reduce or eliminate the increased risk of death associated with prolonged sitting time [27].

To the best of our knowledge, there are no studies on LTPA and sedentary behavior trends among adults with obesity based on the current Korean population. Therefore, this study aimed to identify the 8-year trends in obesity, sedentary behavior, and LTPA. Moreover, it presents the adherence rate of physical activity stratified by obesity status in the Korean population.

## Materials and methods

### Data sources and study population

The Korea National Health and Nutritional Examination Survey (KNHANES) is an ongoing, cross-sectional, nationwide survey on Korean health levels, health-related consciousness and behavior, and nutrition that calculates national statistics every year since 1998. The survey was conducted by the Korea Disease Control and Prevention Agency (KDCA). Furthermore, detailed guidelines and designs have been specified in previous studies. It comprises a stratified, multistage probability sample of the non-institutionalized Korean population designed by trained professionals [28]. This study used data from the KNHANES database from 2014 to 2021. During this period, the same questionnaire was used to measure the physical activity levels. In the present study, the analytical population initially included adults over 19 years of age (N = 50,001). Individuals with missing covariate values or invalid data on physical activity levels and obesity status were excluded from the analysis. There were 42,676 participants included in the final analysis. This study was approved by the Research Ethics Review Board of the KDCA. The current study was approved for exemption by the Institutional Review Board (IRB) of Seoul National University (IRB NO. E2211/004-003).

### Measurements

Trained personnel conducted either face-to-face or self-administered health interviews and examinations at a mobile examination center. Sociodemographic characteristics included age (19–29, 30–39, 40–49, 50–59, 60–69, and ≥70 years), sex (male and female), family income (low, mid-low, mid-high, and high), and educational level (<high school, high school graduate, and ≥college). The anthropometric measurements included height, weight, and waist circumference (WC). BMI was calculated as weight in kilograms divided by the square of the height in meters. Based on their BMI, individuals were categorized as underweight (<18.5 kg/m^2^), normal (≥18.5-<23 kg/m^2^), overweight (≥23-<25 kg/m^2^), or obese (≥25 kg/m^2^) by Korean standards [29]. Abdominal obesity was defined as a WC of ≥ 85 centimeters (cm) in women and ≥90 cm in men [30].

Since 2014, physical activity levels in the KNHANES have been self-reported by individuals using the Global Physical Activity Questionnaire (GPAQ). Physical activity is measured using the GPAQ in terms of intensity, duration, and frequency. In addition, it assesses three domains of physical activity (e.g., physical activity at work, transportation, and leisure) [31, 32]. The GPAQ asks how many times per week, how many hours, and how many minutes of physical activity lasts per domain for more than 10 minutes. Moderate LTPA was defined as 4.0 metabolic equivalent (MET) and continuous physical activity lasting at least 10 min while causing slight breathing or heart rate increases. Vigorous LTPA was defined as 8.0 MET and a continuous 10-minute physical activity that causes a significant increase in breathing or heart rate. Physical activity guidelines recommend that an individual participates in at least 150, 75, and 150 minutes per week of moderate-intensity, vigorous-intensity, and combined moderate-to-vigorous-intensity physical activity, respectively. In this study, we confirmed that only LTPA met these criteria [33, 34].

For sedentary behavior, participants were asked how much time per day they spent sitting or lying down when working, at home, moving to places, or with friends, except when sleeping. The prevalence of sedentary behavior for more than 8 hours per day has been confirmed [35, 36]. According to physical activity guidelines, sleep time was considered to be 8 hours/day of sitting. If the total sitting time was more than 16 hours per day, the data were treated as invalid [37].

### Statistical analysis

All statistical analyses were performed using the SVYDESIGN module of R version 4.1.3 to account for the stratified, multistage probability, and sampling design. We used survey-weighted generalized linear models with the survey cycle included as an independent variable to test linear trends in the prevalence of obesity, sedentary behavior, and adherence to LTPA levels over time. The survey sample weights provided by KNHANES were applied in all analyses to generate estimates that accurately represented the non-institutionalized civilian Korean population. These sampling weights were derived considering the intricate sample design, the non-response rate among the target population, and post-stratification and included unequal probabilities of selection, oversampling, and non-response such that inferences could be made about Korean adult participants. Multivariable logistic regression adjusted for covariates was used to examine the adherence trends to LTPA and the prevalence trends of obesity and sitting time, as well as the interaction between different strata of characteristic variables and the survey year. In addition, we used joinpoint regression to examine changes in trends over time. Prevalence and adherence were expressed as weighted percentages of categorical variables with 95% confidence intervals (CIs), and statistical significance was set at a two-sided *p*-value of <0.05.

## Results

**Fig 1:**
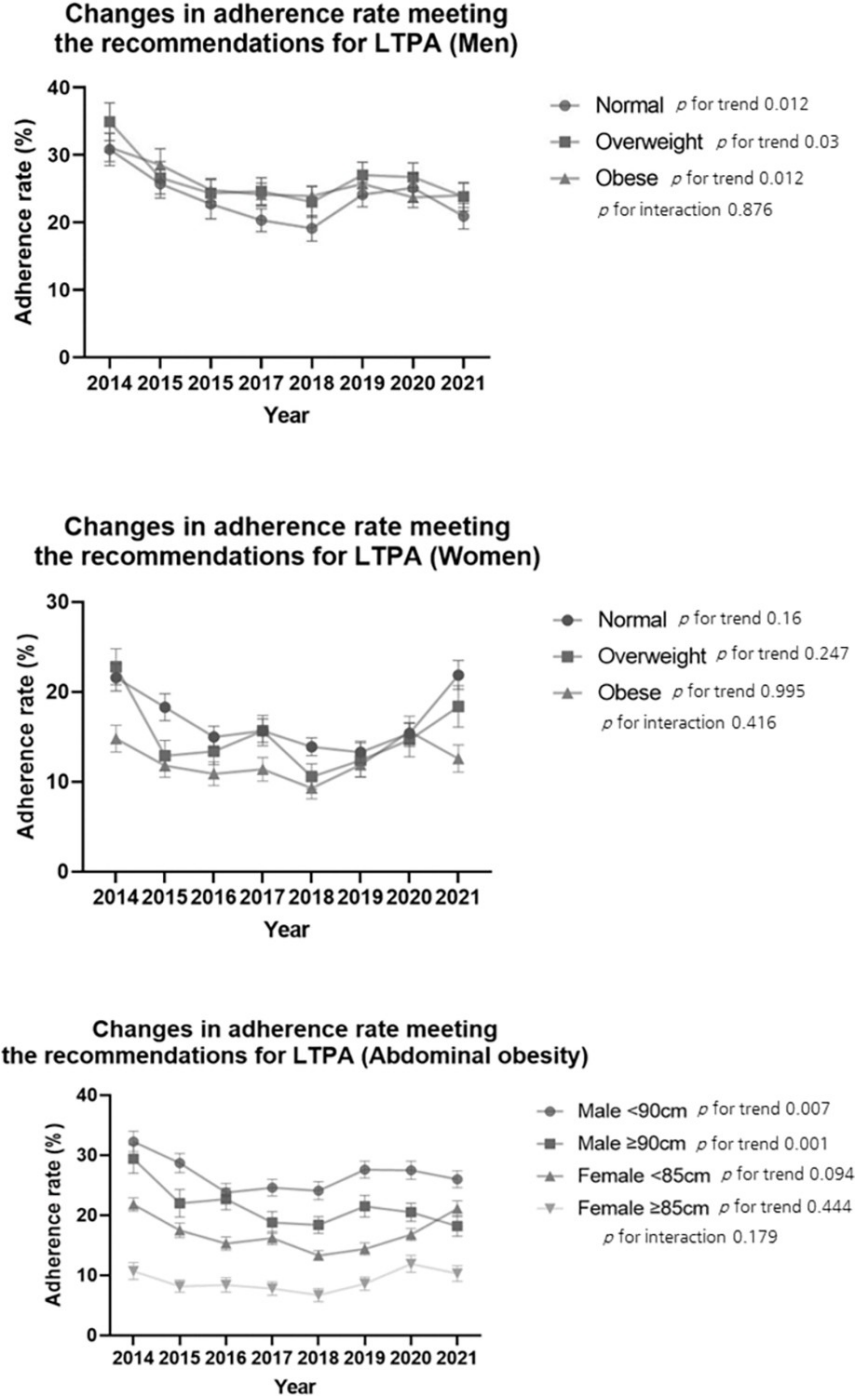
Changes in adherence rate to meeting the recommendations for leisure time physical activity by obesity. The generalized linear model incorporates the Korea National and Nutrition Examination Survey cycle as a continuous variable, adjusted for age, sex, education level, family income, obesity level, waist circumference level, and sedentary behavior level except for the stratified variables.

The baseline characteristics of the study are shown in **(Table 1)**. We analyzed 42,676 participants aged 19 years or older (18759 [44.0%] males; 23917 [56.0%] females) from the KNHANES database between 2014 and 2021. The prevalence of obesity according to BMI in the total age group significantly increased from 30.8% (95% CI, 29.3%-32.7%) in 2014 to 37.3% (95% CI, 35.5%-39.1%) in 2021. We observed that the prevalence of obesity in adult men increased significantly in recent years to 45.0% (95% CI, 42.5%-47.5%) **(Table 2)**. The trend of abdominal obesity, as confirmed by the WC, showed a more significant increase than that of obesity measured by BMI. For both men and women, the abdominal obesity rate increased significantly from 26.4% (95% CI, 24.1%-28.6%) and 19.7% (95% CI, 17.8%-21.7%), respectively, in 2014 to 40.4% (95% CI, 37.6%-43.2%) and 28.8% (95% CI, 26.4%-31.1%), respectively, in 2021. We observed a significantly increased prevalence in several subgroups according to family income and educational level **(Table 3)**. Across all age groups, the proportion of individuals with high sedentary behavior increased significantly (*p* < 0.001). This level was high in their 20s and 70 years of age or older **(Table 4)**. LTPA significantly decreased from 25.5% (95% CI, 23.7%-27.2%) in 2014 to 20.5% (95% CI, 18.7%-22.2%) in 2021. Notably, elderly individuals aged 70 years or older have shown a < 10% adherence rate since 2015. Additionally, we confirmed that individuals with lower income and education levels tended to have lower rates of LTPA adherence **(Table 5)**. In contrast, there have been no differences in the adherence rate of LTPA in the overweight and obese male groups compared to that in the normal weight group in recent years. However, there was a difference in the LTPA adherence rate between obese and normal adults, corresponding to WC. Particularly, women with abdominal obesity have recently increased their LTPA adherence rates to 6.7% (95% CI, 4.6%-8.8%) in 2018, 8.6% (95% CI, 6.4%-10.7%) in 2019, and 11.9% **(Fig 1)**. In addition, we confirmed that LTPA adherence increased again from 2016 through joinpoint regression **(S1 Fig**).

**Table 1:** Baseline characteristics of participants^a^.

	N. (%) of participants								
	Total	2014	2015	2016	2017	2018	2019	2020	2021
**Overall**	42,676	4824	4,886	5628	5632	5749	5723	5197	5037
Age(years)									
19–29	5378 (12.6)	568 (11.8)	632 (12.9)	653 (11.6)	688 (12.2)	732 (12.7)	708 (12.4)	758 (14.6)	639 (12.7)
30–39	6436 (15.1)	850 (17.6)	681 (13.9)	1023 (18.2)	833 (14.8)	867 (15.1)	866 (15.1)	717 (13.8)	599 (11.9)
40–49	7749 (18.2)	838 (17.4)	874 (17.9)	1068 (19.0)	1049 (18.6)	1071 (18.6)	1056 (18.5)	900 (17.3)	893 (17.7)
50–59	8309 (19.5)	948 (19.7)	1032 (21.1)	1039 (18.5)	1131 (20.1)	1122 (19.5)	1109 (19.4)	990 (19.0)	938 (18.6)
60–69	7841 (18.4)	863 (17.9)	904 (18.5)	949 (16.9)	1013 (18.0)	1037 (18.0)	1037 (18.1)	1015 (19.5)	1023 (20.3)
70+	6963 (16.3)	757 (15.7)	763 (15.6)	896 (15.9)	918 (16.3)	920 (16.0)	947 (16.5)	817 (15.7)	945 (18.8)
**Sex**									
Male	18759 (44.0)	2107 (43.7)	2120 (43.4)	2430 (43.2)	2502 (44.4)	2532 (44.0)	2549 (44.5)	2359 (45.4)	2250 (44.7)
Female	23917 (56.0)	2807 (58.2)	2766 (56.6)	3198 (56.8)	3130 (55.6)	3217 (56.0)	3174 (55.5)	2838 (54.6)	2787 (55.3)
**Family income**									
Low	7654 (17.9)	876 (18.2)	884 (18.1)	1045 (18.6)	1058 (18.8)	1015 (17.7)	1039 (18.2)	838 (16.1)	899 (17.8)
Mid-low	10454 (24.5)	1209 (25.1)	1210 (24.8)	1391 (24.7)	1353 (24.0)	1393 (24.2)	1479 (25.8)	1228 (23.6)	1191 (23.6)
Mid-high	11853 (27.8)	1406 (29.1)	1351 (27.7)	1562 (27.8)	1557 (27.6)	1600 (27.8)	1473 (25.7)	1494 (28.7)	1410 (28.0)
High	12715 (29.8)	1333 (27.6)	1441 (29.5)	1630 (29.0)	1664 (29.5)	1741 (30.3)	1732 (30.3)	1637 (31.5)	1537 (30.5)
**Educational Level**									
<High school	12590 (29.5)	1647 (34.1)	1620 (33.2)	1745 (31.0)	1715 (30.5)	1616 (28.1)	1536 (26.8)	1320 (25.4)	1391 (27.6)
High school graduate	14326 (33.6)	1609 (33.4)	1679 (34.4)	1810 (32.2)	1750 (31.1)	1949 (33.9)	1940 (33.9)	1858 (35.8)	1731 (34.4)
> = College	15760 (36.9)	1568 (32.5)	1587 (32.5)	2073 (36.8)	2167 (38.5)	2184 (38.0)	2247 (39.3)	2019 (38.8)	1915 (38.0)

^a^ Data are expressed as unweighted number of participants (n) and percentage (%)

**Table 2:** Trends in prevalence of obesity by body mass index (kg/m^2^) (%, [95% CI]).

	2014	2015	2016	2017	2018	2019	2020	2021	*p* for trend^a^	*p* for interaction^b^
**Overall**	30.8 (29.1–32.5)	33.4 (31.8–35.1)	35.3 (33.5–37.1)	34.5 (32.9–36.2)	35.2 (33.6–36.7)	34.2 (32.7–35.7)	38.8 (37.1–40.4)	37.3 (35.5–39.1)	0.004	
**Age(years)**										
19–29	22.2 (18.0–26.3)	23.7 (19.9–27.5)	27.0 (22.7–31.3)	30.5 (26.5–34.4)	27.1 (23.1–31.1)	27.6 (23.8–31.4)	33.0 (29.5–36.5)	27.8 (23.7–32.0)	0.175	
30–39	31.3 (28.0–34.6)	30.4 (26.4–34.3)	34.4 (31.2–37.6)	32.9 (29.0–36.8)	37.8 (33.6–41.9)	35.0 (31.3–38.7)	41.7 (37.4–46.0)	39.4 (35.4–43.4)	0.764	
40–49	30.7 (27.6–33.8)	34.8 (31.1–38.5)	38.6 (35.3–41.9)	34.9 (31.1–38.7)	36.4 (33.1–39.7)	35.1 (32.1–38.1)	38.7 (34.9–42.6)	43.2 (39.1–47.3)	0.746	
50–59	34.5 (31.5–37.6)	38.4 (35.0–41.9)	35.9 (32.0–39.8)	37.4 (34.3–40.6)	35.8 (32.6–39.1)	36.3 (33.1–39.5)	40.7 (37.2–44.3)	37.2 (33.4–40.9)	0.001	
60–69	36.9 (33.6–40.3)	40.8 (36.9–44.8)	40.0 (36.0–43.9)	37.7 (34.6–40.7)	37.1 (33.8–40.3)	36.8 (33.3–40.3)	41.6 (37.7–45.4)	40.9 (37.0–44.7)	<0.001	
70+	32.5 (28.6–36.3)	36.1 (31.7–40.5)	37.7 (34.1–41.2)	34.1 (30.7–37.6)	38.7 (34.8–42.5)	34.8 (31.1–38.5)	35.8 (32.1–39.6)	33.9 (30.2–37.5)	<0.001	<0.001
**Sex**										
Male	37.1 (34.7–39.5)	38.9 (36.4–41.4)	41.6 (39.1–44.1)	41.2 (39.0–43.4)	42.5 (40.1–44.9)	41.4 (39.2–43.7)	47.4 (45.0–49.9)	45.0 (42.5–47.5)	0.048	
Female	24.8 (22.6–26.9)	28.2 (26.2–30.2)	29.1 (27.0–31.2)	27.9 (25.8–30.0)	27.9 (26.0–29.7)	27.0 (25.0–29.0)	29.9 (27.7–32.1)	29.4 (27.1–31.8)	0.064	0.365
**Family income**										
Low	32.5 (28.4–36.5)	39.6 (35.6–43.6)	37.6 (33.2–42.0)	37.3 (33.1–41.4)	35.6 (31.9–39.4)	33.8 (30.5–37.2)	39.5 (35.2–43.7)	38.1 (33.6–42.6)	0.007	
Mid-low	32.4 (29.2–35.7)	34.8 (31.5–38.0)	36.1 (33.0–39.3)	36.2 (32.9–39.4)	36.2 (33.3–39.1)	35.5 (32.4–38.6)	39.6 (36.1–43.2)	40.3 (36.2–44.3)	0.022	
Mid-high	33.8 (30.5–37.0)	32.0 (28.7–35.2)	35.7 (33.1–38.4)	34.2 (31.2–37.2)	36.9 (33.9–39.9)	35.2 (32.4–38.1)	40.0 (37.1–42.9)	37.1 (34.2–40.1)	0.401	
High	25.7 (22.6–28.8)	30.9 (28.0–33.9)	33.1 (30.0–36.1)	32.4 (29.7–35.1)	32.5 (29.8–35.1)	32.5 (29.6–35.4)	36.9 (34.2–39.5)	35.2 (32.3–38.0)	0.651	0.423
**Educational Level**										
<High school	36.1 (33.4–38.9)	41.9 (38.9–44.9)	41.1 (38.2–43.9)	40.1 (37.5–42.7)	38.6 (35.6–41.6)	40.2 (36.9–43.4)	40.0 (37.0–43.0)	42.1(39.1–35.2)	<0.001	
High school graduate	30.6 (27.7–33.5)	30.7 (27.9–33.5)	33.2 (30.3–36.1)	34.4 (31.6–37.3)	34.5 (31.8–37.1)	33.2 (30.9–35.5)	38.2 (35.4–41.0)	37.8 (35.0–40.5)	0.249	
≥ College	27.4 (24.6–30.1)	30.7 (28.2–33.3)	33.8 (31.5–36.0)	31.7 (29.2–34.3)	34.0 (31.9–36.1)	32.3 (30.2–34.5)	38.7 (36.5–41.0)	34.8 (32.1–37.5)	0.515	0.423

Abbreviation: KNHANES; Korea National and Nutrition Examination Survey.

Defined as obesity by body mass index (≥25kg/m^2^)

^a^ The generalized linear model includes the KNHANES cycle as a continuous variable, adjusted for age, sex, education level, family income, obesity level, waist circumference level, and sedentary behavior level except for the stratified variables.

^b^ The *p*-value for the interactions among different subgroups and the KNHANES cycle is adjusted for all covariates.

**Table 3:** Trends in prevalence of abdominal obesity (%, [95% CI]).

	2014	2015	2016	2017	2018	2019	2020	2021	*p* for trend^a^	*p* for interaction^b^
**Overall**	23.0 (21.4–24.6)	29.0 (27.5–30.4)	29.4 (27.6–31.3)	27.3 (25.6–29.0)	28.0 (26.6–29.5)	33.8 (32.1–35.6)	37.0 (35.5–38.6)	34.6 (32.6–36.7)	<0.001	
**Age(years)**										
19–29	12.7 (9.5–15.9)	16.2 (12.9–19.6)	16.4 (12.9–19.9)	20.4 (16.7–24.0)	14.5 (11.6–17.4)	18.4 (14.7–22.1)	23.3 (20.3–26.3)	18.0 (14.8–21.2)	0.166	
30–39	22.2 (19.0–25.3)	24.8 (21.1–28.5)	28.1 (25.0–31.2)	24.0 (20.3–27.7)	27.7 (24.2–31.1)	28.8 (25.8–31.8)	34.4 (30.6–38.2)	35.2 (30.9–39.5)	<0.001	
40–49	22.3 (19.5–25.2)	27.9 (24.5–31.3)	30.2 (27.0–33.5)	24.2 (21.1–27.4)	26.9 (23.9–30.0)	30.4 (27.5–33.4)	36.0 (32.5–39.5)	34.6 (30.4–38.8)	<0.001	
50–59	23.6 (20.4–26.7)	32.7 (29.6–35.7)	29.2 (25.8–32.7)	27.8 (24.8–30.7)	27.6 (24.5–30.8)	37.4 (34.1–40.8)	39.0 (35.6–42.4)	33.3 (29.7–37.0)	<0.001	
60–69	33.2 (29.5–36.8)	38.3 (34.0–42.5)	37.0 (33.1–40.9)	36.0 (32.3–39.7)	37.4 (34.1–40.7)	43.9 (40.3–47.4)	45.7 (41.7–49.7)	44.8 (41.0–48.7)	<0.001	
70+	34.4 (30.2–38.6)	45.6 (41.1–50.1)	44.6 (40.9–48.3)	39.1 (35.1–43.1)	43.5 (39.3–47.6)	54.2 (50.2–58.1)	52.4 (48.6–56.1)	48.4 (44.3–52.5)	<0.001	<0.001
**Sex**										
Male	26.4 (24.1–28.6)	31.8 (29.8–33.7)	31.2 (28.8–33.5)	32.1 (30.0–34.2)	33.4 (31.2–35.6)	38.4 (36.0–40.8)	44.4 (42.2–46.6)	40.4 (37.6–43.2)	<0.001	
Female	19.7 (17.8–21.7)	26.3 (24.5–28.2)	27.7 (25.5–29.9)	22.5 (20.3–24.6)	22.7 (21.0–24.4)	29.3 (27.2–31.5)	29.4 (27.1–31.8)	28.8 (26.4–31.1)	<0.001	<0.001
**Family income**										
Low	30.7 (26.7–34.7)	38.7 (34.8–42.7)	38.3 (33.5–43.1)	34.4 (30.7–38.2)	35.1 (31.2–39.0)	41.6 (37.6–45.5)	44.5 (40.2–48.8)	42.9 (38.0–47.9)	<0.001	
Mid-low	24.9 (22.1–27.7)	31.2 (28.2–34.3)	30.6 (27.9–33.4)	30.3 (27.1–33.4)	30.0 (27.6–32.5)	36.6 (33.4–39.8)	39.5 (35.7–43.4)	41.2 (36.8–45.6)	<0.001	
Mid-high	23.0 (20.3–25.7)	27.5 (24.9–30.2)	29.2 (26.6–31.7)	25.7 (22.7–28.6)	27.0 (24.3–29.7)	32.1 (29.3–35.0)	35.0 (32.1–37.9)	33.2 (30.5–35.9)	<0.001	
High	18.0 (15.4–20.7)	24.2 (21.7–26.7)	24.4 (21.6–27.2)	23.3 (20.7–25.9)	24.1 (21.9–26.3)	29.8 (26.6–33.0)	34.4 (32.1–36.6)	28.6 (25.7–31.4)	<0.001	0.268
**Educational Level**										
<High school	31.3 (28.6–33.9)	41.0 (38.0–43.9)	40.3 (37.3–43.2)	37.7 (35.0–40.4)	37.9 (34.9–40.9)	50.3 (47.3–53.3)	49.3 (45.7–53.0)	48.8 (45.4–52.2)	<0.001	
High school graduate	21.0 (18.7–23.3)	25.7 (23.2–28.2)	25.4 (22.8–28.0)	26.5 (23.6–29.4)	25.6 (23.2–28.1)	31.7 (29.3–34.0)	35.6 (32.7–38.4)	33.5 (30.6–36.4)	<0.001	
≥ College	19.4 (17.1–21.8)	24.5 (22.1–26.9)	26.7 (24.1–29.3)	22.4 (20.2–24.6)	25.1 (23.1–27.1)	28.3 (26.1–30.4)	33.1 (30.8–35.4)	29.6 (26.8–32.3)	<0.001	0.01

Abbreviation: KNHANES; Korea National and Nutrition Examination Survey.

Defined as abdominal obesity by waist circumference (Male≥90cm; Female≥85cm).

^a^ The generalized linear model includes the KNHANES cycle as a continuous variable, adjusted for age, sex, education level, family income, obesity level, waist circumference level, and sedentary behavior level except for the stratified variables.

^b^ The *p*-value for the interactions among different subgroups and the KNHANES cycle is adjusted for all covariates.

**Table 4:** Changes in adherence rate meeting the recommendations for leisure time physical activity (%, [95% CI]).

	2014	2015	2016	2017	2018	2019	2020	2021	*p* for trend^a^	*p* for interaction^b^
**Overall**	25.5 (23.7–27.2)	20.7 (18.9–22.5)	18.4 (16.7–20.1)	18.5 (16.9–20.1)	17.0 (15.5–18.4)	18.9 (17.3–20.6)	19.9 (18.5–21.4)	20.5 (18.7–22.2)	<0.001	
Age(years)										
19–29	32.2 (27.5–36.8)	30.6 (26.4–34.8)	28.0 (23.3–32.6)	27.2 (23.2–31.2)	26.5 (22.5–30.5)	28.8 (24.3–33.4)	30.2 (26.4–34.0)	33.6 (29.4–37.7)	0.759	
30–39	23.2 (19.8–26.6)	20.3 (16.8–23.8)	15.3 (12.6–18.1)	20.0 (16.4–23.6)	19.0 (15.8–22.2)	22.6 (18.8–26.4)	21.1 (18.0–24.2)	23.6 (19.6–27.6)	0.347	
40–49	28.7 (25.3–32.1)	24.4 (21.0–27.9)	23.1 (19.9–26.3)	21.1 (17.9–24.4)	16.6 (13.9–19.2)	19.4 (16.4–22.4)	18.7 (15.9–21.6)	21.1 (17.6–24.5)	<0.001	
50–59	28.7 (25.4–32.0)	18.6 (15.0–22.1)	16.7 (14.1–19.4)	18.1 (15.1–21.0)	15.5 (12.9–18.2)	16.3 (13.4–19.2)	17.5 (14.2–20.8)	17.9 (14.9–21.0)	<0.001	
60–69	17.1 (13.9–20.3)	14.2 (11.1–17.3)	14.6 (11.7–17.4)	11.7 (9.2–14.1)	13.1 (10.8–15.4)	14.2 (11.3–17.0)	17.1 (14.6–19.7)	14.9 (12.1–17.8)	0.398	
70+	11.8 (9.2–14.4)	6.0 (3.8–8.1)	5.4 (3.7–7.0)	5.1 (3.0–7.2)	5.3 (3.6–7.1)	6.8 (4.6–9.1)	9.9 (7.3–12.5)	5.9 (4.3–7.6)	0.526	<0.001
**Sex**										
Male	31.5 (28.8–34.3)	26.6 (23.8–29.3)	23.5 (21.1–25.9)	22.7 (20.5–25.0)	22.2 (19.9–24.4)	25.2 (22.9–27.5)	24.4 (22.4–26.5)	22.9 (20.6–25.1)	<0.001	
Female	19.6 (17.6–21.6)	15.1 (13.2–16.9)	13.4 (11.7–15.1)	14.3 (12.5–16.1)	11.8 (10.4–13.2)	12.7 (11.0–14.4)	15.3 (13.6–17.1)	18.0 (15.9–20.2)	<0.001	<0.001
**Family income**										
Low	14.3 (11.1–17.4)	9.9 (7.2–12.5)	8.5 (5.8–11.2)	8.3 (5.9–10.7)	9.2 (6.2–12.2)	10.1 (7.5–12.7)	9.9 (7.4–12.5)	9.6 (7.0–12.2)	0.163	
Mid-low	21.9 (18.2–25.5)	16.7 (13.6–19.8)	13.0 (10.6–15.4)	14.5 (12.0–17.0)	12.7 (10.6–14.8)	13.4 (11.2–15.7)	16.4 (13.6–19.2)	16.8 (13.8–19.8)	0.182	
Mid-high	28.1 (25.1–31.1)	21.0 (17.7–24.3)	19.1 (16.8–21.5)	18.8 (16.0–21.6)	16.6 (14.0–19.2)	20.6 (17.4–23.8)	21.1 (18.5–23.6)	21.4 (18.0–24.8)	0.104	
High	30.8 (27.8–33.8)	28.4 (25.3–31.4)	26.6 (23.7–29.6)	25.8 (23.0–28.7)	24.4 (21.8–26.9)	25.5 (22.9–28.1)	24.8 (21.9–27.7)	26.1 (23.8–28.4)	0.009	0.351
**Educational Level**										
<High school	13.5 (11.3–15.7)	7.2 (5.5–8.9)	5.6 (4.2–7.1)	6.2 (4.5–7.8)	6.6 (4.9–8.2)	7.7 (5.7–9.8)	7.7 (6.1–9.4)	7.9 (6.1–9.7)	0.173	
High school graduate	27.6 (24.8–30.4)	23.1 (20.2–26.0)	20.1 (17.5–22.6)	19.2 (16.8–21.6)	17.3 (15.2–19.4)	19.0 (16.5–21.5)	21.2 (18.8–23.5)	19.7 (17.2–22.3)	0.003	
≥ College	31.4 (28.5–34.3)	26.9 (23.9–30.0)	24.5 (22.0–27.0)	24.4 (22.0–26.9)	22.0 (19.7–24.3)	23.9 (21.4–26.3)	23.9 (21.7–26.1)	26.5 (23.8–29.2)	0.073	0.219

Abbreviation: KNHANES; Korea National and Nutrition Examination Survey.

Defined as leisure-time physical activity (moderate-to-vigorous physical activity ≥ 150 minutes/week)

^a^ The generalized linear model includes the KNHANES cycle as a continuous variable, adjusted for age, sex, education level, family income, obesity level, waist circumference level, and sedentary behavior level except for the stratified variables.

^b^ The *p*-value for the interactions among different subgroups and the KNHANES cycle is adjusted for all covariates.

**Table 5:** Trends in prevalence of sedentary behavior (%, [95% CI]).

	**2014**	**2015**	**2016**	**2017**	**2018**	**2019**	**2020**	**2021**	***p* for trend** ^**a**^	***p* for interaction** ^**b**^
**Overall**	46.7 (44.4–49.0)	52.1 (50.0–54.3)	53.6 (51.7–55.5)	56.2 (54.4–58.0)	57.9 (55.9–59.9)	59.7 (57.9–61.6)	59.2 (57.1–61.2)	63.0 (60.7–65.3)	<0.001	
Age(years)										
19–29	61.1 (56.9–65.2)	67.2 (63.3–71.2)	67.2 (62.4–72.1)	71.6 (67.7–75.5)	74.2 (70.5–77.9)	70.8 (66.8–74.8)	72.0 (68.5–75.6)	75.1 (70.9–79.4)	<0.001	
30–39	46.9 (42.2–51.5)	54.7 (49.9–59.5)	56.4 (52.8–60.0)	62.4 (58.7–66.1)	61.9 (57.9–65.9)	61.6 (57.4–65.8)	62.4 (58.0–66.9)	65.0 (60.0–69.9)	<0.001	
40–49	45.8 (41.6–50.0)	49.7 (45.4–54.1)	49.8 (46.6–53.0)	53.1 (49.4–56.8)	55.8 (52.0–59.6)	59.6 (55.7–63.5)	57.7 (53.5–61.8)	60.8 (56.7–64.8)	<0.001	
50–59	40.7 (36.5–44.9)	43.2 (39.3–47.1)	46.7 (43.3–50.1)	46.0 (42.4–49.6)	45.8 (42.1–49.5)	51.5 (48.1–54.8)	51.2 (46.9–55.5)	52.5 (48.2–56.8)	<0.001	
60–69	35.1 (30.9–39.3)	38.6 (34.3–42.9)	44.2 (40.3–48.0)	43.5 (39.5–47.4)	46.4 (42.4–50.5)	53.3 (49.7–57.0)	49.8 (45.4–54.2)	57.1 (53.3–60.9)	<0.001	
70+	46.0 (41.0–51.0)	57.6 (53.4–61.8)	57.9 (54.1–61.7)	60.9 (56.8–65.0)	64.0 (60.0–68.0)	62.5 (58.3–66.7)	63.2 (59.0–67.4)	72.0 (68.5–75.4)	<0.001	0.084
**Sex**										
Male	47.9 (44.6–51.1)	52.5 (49.8–55.3)	55.3 (52.6–58.0)	57.1 (54.7–59.5)	58.9 (56.2–61.6)	60.3 (57.8–62.8)	59.8 (56.9–62.6)	63.7 (60.8–66.6)	<0.001	
Female	45.6 (43.1–48.2)	51.8 (49.0–54.5)	51.9 (49.6–54.2)	55.3 (53.1–57.6)	56.8 (54.5–59.1)	59.2 (57.1–61.3)	58.5 (56.4–60.7)	62.3 (59.7–64.8)	<0.001	0.971
**Family income**										
Low	43.8 (39.1–48.5)	49.8 (45.1–54.5)	54.1 (49.7–58.5)	56.7 (53.1–60.3)	55.0 (50.8–59.3)	60.6 (56.9–64.3)	61.3 (57.3–65.4)	69.1 (64.8–73.5)	<0.001	
Mid-low	40.3 (36.8–43.8)	45.6 (41.9–49.4)	48.6 (45.3–51.9)	48.7 (45.0–52.3)	53.2 (49.6–56.9)	53.2 (50.1–56.3)	55.0 (51.2–58.8)	56.3 (52.6–60.1)	<0.001	
Mid-high	45.6 (42.0–49.2)	51.3 (48.0–54.7)	51.1 (48.2–54.0)	55.8 (53.0–59.6)	57.6 (54.5–60.7)	58.1 (54.7–61.4)	56.7 (53.7–59.7)	61.5 (57.8–65.1)	<0.001	
High	54.5 (50.4–58.5)	58.8 (55.1–62.5)	59.4 (56.2–62.6)	61.8 (58.4–65.1)	63.1 (60.1–66.1)	65.7 (62.5–68.9)	63.1 (59.7–66.4)	66.5 (63.2–69.8)	<0.001	0.604
**Educational Level**										
<High school	36.7 (33.0–40.3)	42.0 (38.7–45.3)	46.8 (43.7–49.9)	45.5 (42.2–48.9)	50.6 (47.2–54.0)	53.1 (49.7–56.5)	52.8 (48.9–56.6)	57.8 (54.5–61.1)	<0.001	
High school graduate	44.0 (40.8–47.2)	51.0 (47.9–54.1)	51.7 (48.5–54.8)	52.4 (49.2–55.6)	54.2 (51.6–56.7)	56.1 (53.4–58.7)	56.0 (53.1–59.0)	60.7 (57.3–64.2)	<0.001	
≥ College	56.5 (53.5–59.5)	59.9 (56.7–63.0)	59.4 (56.9–61.9)	64.7 (62.3–67.2)	64.9 (62.0–67.7)	65.7 (63.0–68.3)	64.7 (61.6–67.8)	67.2 (64.4–70.0)	<0.001	0.396

Abbreviation: KNHANES; Korea National and Nutrition Examination Survey.

Defined as sedentary behavior (sitting time ≥ 8 hours/day)

^a^ The generalized linear model includes the KNHANES cycle as a continuous variable, adjusted for age, sex, education level, family income, obesity level, waist circumference level, and sedentary behavior level except for the stratified variables.

^b^ The *p*-value for the interactions among different subgroups and the KNHANES cycle is adjusted for all covariates.

## Discussion

In this nationally representative Korean study, we found that the proportion of people with obesity and sedentary behavior increased significantly from 2014 to 2021. Although there was a significant trend among all adults, the joinpoint analysis confirmed the trend of increasing again in 2016 as an inflection point. However, the overall adherence rate remained low. Abdominal obesity showed a more significant trend by year than the obesity level as measured by BMI. In particular, the obesity rate was the highest when there was a lockdown due to COVID-19 in 2020. To be specific, there was a significant difference in the LTPA level according to income and education level, and the overall adherence rate was also low. In addition, adherence to LTPA differed according to the obesity level. Sedentary behavior has increased significantly annually since 2014. Furthermore, it was observed that this behavior was most prominent among individuals in their 20s and upper 70s. The high sedentary behavior among older adults is consistent with trends observed in previous studies conducted in other countries [12, 38, 39].

From 2004 to 2018 in China, there was an increase in the standardized mean BMI levels from 22.7 kg/m^2^ to 24.4 kg/m^2^, and the prevalence of obesity increased from 3.1% to 8.1%. The annual increase in mean BMI between 2010 and 2018 was 0.09 kg/m^2^, which was lower than the reported increase of 0.17 kg/m^2^ from 2004 to 2010 [40]. In addition, the prevalence of abdominal obesity increased from 15.4% in 2000 to 29.9% in 2014 among men, and from 12.3% in 2000 to 19.8% in 2014 among women [41]. However, the prevalence of abdominal obesity in South Korea has been shown to be higher and increased rapidly in this study. In a Chinese study that categorized BMI and abdominal obesity to examine physical activity levels, the LTPA adherence rate was higher in the overweight and obese groups, and in the group with abdominal obesity than in those without abdominal obesity. In contrast, a Korean study found no remarkable differences in the BMI categories. However, groups with abdominal obesity were observed lower levels of physical activity than individuals without abdominal obesity. Furthermore, our study analyzed trends in increasing sedentary behavior and assessed physical activity adherence across different obesity categories. Obesity showed a consistent upward trend in both BMI and WC. Recent studies have suggested that the WC is a more helpful indicator than the BMI for predicting chronic diseases and assessing their association with obesity-related health outcomes [42–45]. In this study, the significance of the obesity trend as measured by the WC was more pronounced, suggesting that it could have significant public health and economic implications due to its association with various health outcomes [1, 46].

According to recent studies on physical activity trends in Korean adults, including both occupational and transportation-related activities, the prevalence of meeting the WHO physical activity guidelines for performing at least 150 min of moderate-to-vigorous physical activity per week showed a declining trend, from 57% in 2014 to 45.6% in 2019 [47]. Previous studies on physical activity trends in Korea have examined overall levels of physical activity in daily life but have not separately analyzed LTPA. Consistent with previous studies conducted in other countries, individuals with lower educational and income levels tended to have lower rates of meeting LTPA recommendations [48–50]. Individuals with lower socioeconomic or educational levels are more likely to be at a higher risk of developing abdominal obesity due to the increased consumption of processed foods and lower accessibility to LTPA [51–54]. However, since the onset of the COVID-19 pandemic in 2020, lifestyle patterns across the population have undergone rapid changes, leading to a significant increase in overall obesity rates.

Furthermore, our study also confirmed a slight increase in LTPA during this period. Particularly, we observed that female groups with a normal or overweight BMI and normal waist circumference showed a recent rise in LTPA levels. Prior research reported that approximately 31.9% experienced an increase in exercise frequency during the COVID-19 pandemic period [55]. Moreover, it has been suggested that there is a significant possibility of a new influx of people starting physical activity who had not engaged in physical activity previously. In our study, it is likely to consider the influx of this population group, considering that the LTPA levels in the female group were initially low or absent compared to the male group. Additionally, the limitations on activities such as using the fitness center were compensated by heightened awareness of health management. The emphasis on the significance of physical activity for improving mental health led to more conscientious engagement in its practice [56]. Consequently, it can be inferred that home workouts and outdoor activities have witnessed a notable surge in popularity, leading to an upsurge in the population’s engagement in the management of lifestyle habits through physical activity.

In a systematic review of the increasing trend in sedentary behavior, all 26 surveyed studies reported a recent increase in sedentary lifestyle, indicating a universally prevalent phenomenon worldwide [57]. Korea holds the foremost position in worldwide smartphone and internet usage, and most of the surveyed prominent countries have reported internet and smartphone usage rates exceeding 80% [58]. Furthermore, according to Seo et al. (2022), the average sedentary time was 9.7 hours for those in their 20s and 9.2 hours for those aged 70 years and over in 2020, with an increasing trend observed every year in Korea [12]. The duration of sedentary behavior showed a substantial rise over the years, increasing from an average of 5.7 hours per day in 2007–2008 to 6.4 hours per day in 2015–2016 in US adults (*p* < 0.001) [10]. One study investigated sedentary behavior across different domains such as computer use at home, video game use, reading, etc [59]. Another study revealed that increased sedentary time related to work activities was associated with improved cognitive function, whereas sedentary leisure time showed no significant association [60]. Therefore, it will be necessary to measure sedentary time by differentiating between domains of physical activity and incorporating factors such as telecommuting, leisure time smartphone, and mobile content usage. This approach would help better understand the impact of sedentary behavior and provide more accurate recommendations for reducing sedentary time.

Both prolonged sedentary behavior and physical inactivity are associated with various diseases and a higher risk of mortality, resulting in negative outcomes [33, 61–64]. Although a slight upward trend in LTPA has been observed in recent years, the adherence rate remains at a low level. It is considered crucial to recommend guidelines from a public health and policy perspective, emphasizing the importance of physical activity, including strategies that highlight the health benefits of substituting sedentary behavior with physical activity [65]. These guidelines can not only inform individuals about the positive impacts of physical activity on health but also provide a framework for creating supportive environments and policies that facilitate this behavioral change.

The major strength of this study is that it confirmed the trends of LTPA and sedentary behavior in the Korean population. Second, the LTPA level was confirmed by dividing the patients into subgroups according to the BMI and WC criteria. Third, this study reflected the most recent trends and used accurate and representative data of Korean adults from the KNHANES database. However, this study has several limitations. First, the period from 2014 to 2021 was too short to check trends. Long-term studies using the same measurement tool will be necessary. Second, physical activity and sedentary behavior information were self-reported, which could lead to incorrect reporting and measurement bias. In addition, there may be measurement bias due to the tendency of men to overestimate their physical activity levels in self-reported surveys [66]. Finally, the present study did not measure sedentary behavior by domain. Therefore, future studies should investigate sedentary time in more specific domains, such as work and leisure-related.

## Conclusion

This study showed trends in obesity, sedentary behavior, and LTPA in Korean adults from 2014 to 2021. The results indicate a significant increase in both obesity rates and the amount of sedentary behavior, alongside a decrease in adherence to LTPA. Particularly, adherence to LTPA was found to be low among women with obesity. Future research is needed to examine the trends in objective physical activity levels and adherence rates in other areas. In addition, studies should be conducted to evaluate the effectiveness of various interventions for promoting physical activity and reducing sedentary behavior.

## Supporting information

S1 Fig Trends in LTPA index: Joinpoint analysis. (DOCX)
