# Supplementary material for: Trends in obesity, leisure-time physical activity, and sedentary behavior in Korean adults: Korea national health and nutritional examinations survey from 2014 to 2021
Source: PLoS One. 2024 Jan 3;19(1):e0296042. doi: 10.1371/journal.pone.0296042 (PMC10763961; doi:10.1371/journal.pone.0296042)

**S1 Appendix**

**S1 figure: Trends in LTPA index: joinpoint analysis**

We conducted a joinpoint analysis using the R program, and when analyzing the entire population, the value of psi1.year was 2015.6. Upon plotting, it was evident that a change point occurred around the end of 2015. While there was a slight decrease in 2018, we observed a gradual increasing trend from 2016 onwards.


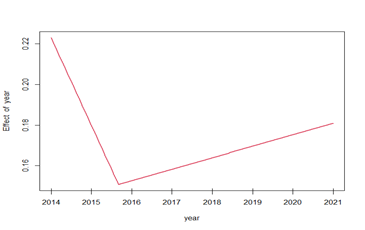

Supplement: S1 Fig — (DOCX) [file pone.0296042.s001.docx]
